# Haematochezia from a Splenic Artery Pseudoaneurysm Communicating with Transverse Colon: A Case Report and Literature Review

**DOI:** 10.1155/2016/8461501

**Published:** 2016-07-31

**Authors:** James O'Brien, Francesca Muscara, Aser Farghal, Irshad Shaikh

**Affiliations:** Department of General Surgery, Norfolk and Norwich University Hospital, Colney Lane, Norwich, Norfolk NR4 7UY, UK

## Abstract

Splenic artery aneurysms (SAA) are the third most common intra-abdominal aneurysm. Complications include invasion into surrounding structures often in association with preexisting pancreatic disease. We describe an 88-year-old female, with no history of pancreatic disease, referred with lower gastrointestinal bleeding. CT angiography showed a splenic artery pseudoaneurysm with associated collection and fistula to the transverse colon at the level of the splenic flexure. The pseudoaneurysm was embolised endovascularly with metallic microcoils. Rectal bleeding ceased. The patient recovered well and follow-up angiography revealed no persistence of the splenic artery pseudoaneurysm. SAA rupture results in 29%–50% mortality. Experienced centres report success with the endovascular approach in haemodynamically unstable patients, as a bridge to surgery, and even on a background of pancreatic disease. This case highlights the importance of prompt CT angiography, if endoscopy fails to identify a cause of gastrointestinal bleeding. Endovascular embolisation provides a safe and effective alternative to surgery, where anatomical considerations and local expertise permit.

## 1. Introduction

Splenic artery aneurysms (SAA) are defined as a ≥1 cm dilatation of the artery diameter and are the third most common intra-abdominal aneurysm [1]. The majority of SAA are detected as incidental findings, but if they present with rupture, a high mortality rate results [2]. Complications include invasion into and communication with surrounding structures, often in association with preexisting pancreatic disease [1, 3]. Traditionally, treatment of SAA was through surgery, but endovascular therapy is now established with minimal morbidity and mortality [4]. We describe successful endovascular management of a splenic artery pseudoaneurysm, with a fistula between the pseudoaneurysm and the transverse colon, in a patient without coexisting pancreatic disease.

## 2. Case Report

An 88-year-old Caucasian female was referred from the emergency department with lower gastrointestinal bleeding. She gave a history of five episodes of fresh rectal bleeding with blood separate from the stools, of one day's duration. This was preceded by one day of loose stools and a constant low grade central abdominal pain. There were no other associated upper or lower gastrointestinal symptoms and no systemic disturbance. Past medical history included atrial fibrillation for which she was prescribed warfarin. She was a nonsmoker with minimal alcohol intake.

On examination, body mass index was 23 and blood pressure 140/70 with an irregular heart rate of 80 bpm, a respiratory rate of 18, and oxygen saturation 100% on room air. Examination of the abdomen elicited tenderness in the periumbilical region with no peritonism. On digital rectal examination the rectum was empty with no masses and no perianal disease. Dark blood was noted on the glove with no clots. Haematological and biochemical investigation revealed haemoglobin was 103 g/L (normal range 115–160 g/L), C-reactive protein 210 mg/L (<10 mg/L), and international normalised ratio 4.47 (target 2.5). Urea and electrolytes, liver function, and serum amylase were normal. Electrocardiogram confirmed atrial fibrillation.

**Figure 1:**
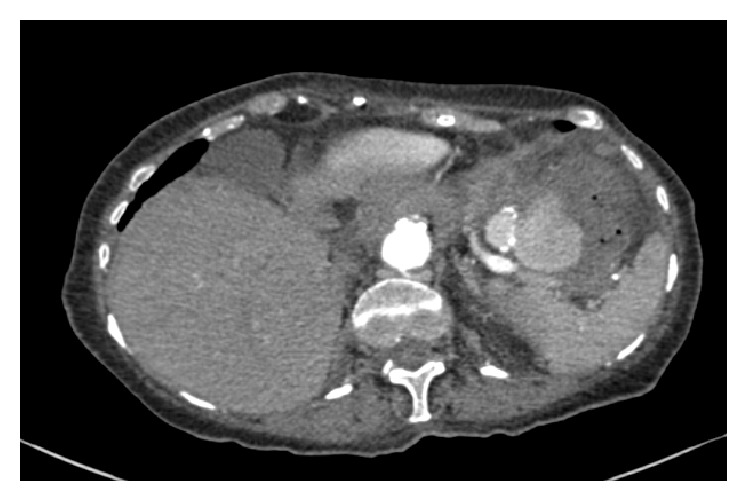
CT angiography axial plane demonstrating splenic artery pseudoaneurysm.

The patient was managed with intravenous fluid replacement and 10 mg vitamin K. Warfarin was stopped. A flexible sigmoidoscopy was performed with no polyps or masses identified and fresh blood and clots seen throughout the sigmoid colon. The patient continued to report bleeding and repeat haemoglobin had decreased to 81 g/L. Two units of packed red cells were transfused and an oesophagogastroduodenoscopy revealed atrophic gastritis and a prepyloric erosion unlikely to be the source of bleeding. Following this computed tomography (CT) angiography showed a splenic artery pseudoaneurysm with associated collection and fistula to the transverse colon at the level of the splenic flexure (Figure 1).

**Figure 2:**
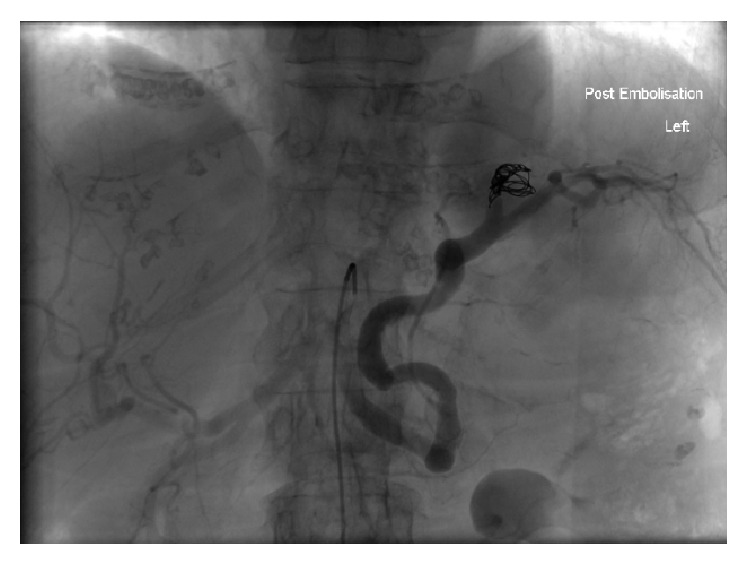
Coeliac arteriography coronal plane demonstrating embolisation of the splenic artery pseudoaneurysm.

Radiology consult was obtained and the pseudoaneurysm embolised via catheter directed metallic microcoils. Upon completion no further contrast extravasation was seen, with cessation of flow through the fistula (Figure 2). Rectal bleeding ceased and there were no complications. The patient recovered well and was discharged. A follow-up CT angiography at 6 weeks after embolisation revealed no persistence of the splenic artery pseudoaneurysm.

## 3. Discussion

Visceral artery aneurysms are rare and reported prevalence in the population varies from 0.1 to 10.4% [5–8]. SAA are the most common type, accounting for 30%–60% [5]. SAA account for up to 60% of all splanchnic artery aneurysms, followed by aneurysms of the hepatic (20%), superior mesenteric (5.9%), and celiac (4%) arteries [9]. Following the aorta and iliac arteries, SAA are the third most common abdominal aneurysm [1, 7].

SAA is a ≥1 cm dilatation of the artery diameter and can be classified as a true or pseudoaneurysm, with the majority (72%) true aneurysms [10]. Most SAA are reported in the main body of the artery, with a majority (74–87%) at the distal third and the mean size 2.1 cm [11–13]. Aneurysm size is not a predictor of rupture [5]. Aetiology is hypothesised as wall degeneration or dilatation of an artery through increased pressure and weakness in the wall [8, 14, 15]. There is increased female : male incidence reported for all SAA [1, 2, 5, 7] and increased male : female incidence reported for giant SAA (defined as a SAA ≥ 5 cm) [2]. Pregnancy, portal hypertension, liver transplant, and pancreatitis are described as particular risk factors, with the latter more closely associated with pseudoaneurysms and the first two factors with true aneurysms [2, 16, 17]. Multiparity has long been associated with increased risk of rupture [1, 7, 18, 19].

20% of SAA are symptomatic and 80% incidental findings [2, 8, 16]. Intervention is recommended for SAA that are symptomatic, increasing in size, found during pregnancy (or in child bearing years), of diameter ≥2 cm (or any size in case of a pseudoaneurysm), as these factors have been described as increasing the risk of rupture [20].

Traditionally 10% of SSA presented with rupture but due to increasing incidental diagnosis this has reduced to 3% [1, 21]. Mortality following rupture was 25% in the 1970s, with little improvement since, and is as high as 100% for pseudoaneurysms [1, 22]. In pregnancy 75% maternal and 95% fetal mortality rates are described. Most aneurysms rupture in this group (95%), with two-thirds during the third trimester [23, 24]. Pathogenesis is hypothesised to be due to the hormonal (oestrogen, progesterone, and relaxin [21, 25, 26]) and physiological changes of pregnancy on the arterial wall and the presence of antenatal comorbidity such as portal hypertension, which is itself a risk factor for SAA [27, 28]. There is an incidence of 7.1–13.0% in this group of patients. Rupture has been described via containment in the lesser sac followed by a second rupture into the greater sac or through a single rupture into the abdomen [26, 29–31]. The symptoms associated with unruptured SAA are usually nonspecific whereas a ruptured SAA almost always presents with hemodynamic instability and severe sudden abdominal pain [1].

Invasion into the stomach, duodenum, pancreatic duct, and colon can result in gastrointestinal bleeding and up to 13% of ruptured SAA have been described as fistulate with these structures [1, 16]. Fistulation to vascular structures such as the splenic and portal veins can cause arteriovenous fistulae resulting in mesenteric steal and small bowel ischaemia [32]. External mass effect on the portal vein can cause portal hypertension and venous congestion [33]. SAA can rupture into pancreatic pseudocysts [20, 34]. 60% of pseudoaneurysms occurring in chronic pancreatitis are SAA [35]. Haemorrhage is described as presenting with a sentinel bleed before major haemorrhage and bleeding as a result of SAA can present with haematemesis, haematochezia, intra-abdominal bleeding, or melaena [1].

This patient presented with rectal bleeding due to fistulation with the transverse colon. Direct colonic involvement presenting with haematochezia without pancreatic involvement is extremely rare in the literature. The first two nonfatal cases of SAA with colonic involvement were reported in 1984 and 2003. In both the SAA communicated with the splenic flexure and required open surgery for definitive treatment [19, 36]. Other authors describe haematochezia from SAA rupturing into a pancreatic pseudocyst with fistula to the colon and in two patients with pancreatitis, a giant pseudoaneurysm communicating with the splenic flexure, and a saccular SAA with a collection extending to the descending colon [3, 37, 38]. Haematochezia from a splenic artery pseudoaneurysm in a patient with a pancreatic pseudocystocolic fistula was successfully treated after the pseudoaneurysm was embolised via catheter directed coils [39]. A patient with chronic pancreatitis underwent successful embolisation of a splenic artery pseudoaneurysm that ruptured into the colon, following a negative laparotomy [40, 41]. The Mayo clinic published an 18-year case series that included a single patient with splenic artery pseudoaneurysm fistulate to the descending colon, without pancreatitis. This patient was treated with surgery. The authors combined their case series with literature review and found 26.2% of splenic pseudoaneurysms present with haematochezia or melaena and in 42% of the 59 patients included, the bleeding originated from the pancreatic duct [20].

Digital subtraction angiography is the preferred modality for delineating SAA and computed tomography for monitoring during conservative management [1]. Endovascular management is now recommended for management of unruptured SAA, including pseudoaneurysms, not involving the splenic hilum [42], through transcatheter embolisation or less commonly stent grafts, with splenic preservation possible [4]. For ruptured SAA or pseudocyst involvement, surgery is recommended [1, 3]. The failure rate of transcatheter embolisation is higher when pseudocyst is present [20]. When a pancreatic pseudocyst is the underlying cause, splenic and pancreatic conserving approaches are described but surgery can be as extensive as aneurysmal resection, splenectomy, and colonic resection with distal pancreatectomy [1]. Ruptured SAA results in high mortality (29%–50%) [20, 43, 44] even following operative management and recent publications advocate endovascular intervention for SAA even on a background of pancreatic disease [45–47]. Experienced centres report success with the endovascular approach even in haemodynamically unstable patients or as a bridge to surgery [44, 48]. Elective laparoscopic approaches have been advocated where loss of splenic function or repeated imaging is contraindicated and where anatomy presents difficulty for embolisation [24, 49]. Zero morbidity or mortality is reported from laparoscopic resection of unruptured SAA [50].

Interventional endovascular treatment for all visceral artery aneurysms is reported with zero mortality for unruptured aneurysms [5, 51]. Although case series for SAA are small, several authors describe zero mortality following endovascular treatment for unruptured SAA since 1987 [43, 44, 52]. Anatomical variation is suggested as the main factor determining successful nonoperative treatment [53]. Aneurysms of the distal artery are more likely to develop complications following endovascular therapy [44]. Recanalisation rates for SAA were quoted as 12.5% in the in 1990s [54]. A large case series in 2015 reports a 93% success rate for all visceral artery aneurysms treated with interventional techniques [5]. Complications of interventional techniques include thrombosis or embolism resulting in organ abscesses and infarction, coil migration, aneurysm recurrence, and local arterial access complications [42]. There is little consensus on follow-up [44].

## 4. Conclusions

Splenic artery aneurysms and pseudoaneurysms are rarely encountered in routine practice but will increasingly be identified as incidental findings. Patients presenting with haematochezia on a background of pancreatic disease should immediately alert the physician to the possibility of splenic artery aneurysm or pseudoaneurysm, complicated by gastrointestinal involvement. Without this history, cases of rectal bleeding caused by SAA or pseudoaneurysm communicating with the colon present a diagnostic challenge. This highlights the importance of prompt CT angiography, especially if upper and lower gastrointestinal endoscopy fail to identify a cause of bleeding. This case describes a splenic artery pseudoaneurysm with direct colonic involvement, in a patient without a background of pancreatic disease, managed successfully without open surgery. Advances in endovascular embolisation techniques provide a safe and effective alternative to surgery, where anatomical considerations and local expertise permit.

## References

[1] Al-Habbal Y., Christophi C., Muralidharan V. (2010). Aneurysms of the splenic artery—a review. *Surgeon*.

[2] Akbulut S., Otan E. (2015). Management of giant splenic artery aneurysm: comprehensive literature review. *Medicine*.

[3] Zhao J., Kong X., Cao D., Jiang L. (2014). Hematochezia from splenic arterial pseudoaneurysm ruptured into pancreatic pseudocyst coexisting with fistula to the colon: a case report and literature review. *Gastroenterology Research*.

[4] Guillon R., Garcier J. M., Abergel A. (2003). Management of splenic artery aneurysms and false aneurysms with endovascular treatment in 12 patients. *CardioVascular and Interventional Radiology*.

[5] Pitton M. B., Dappa E., Jungmann F. (2015). Visceral artery aneurysms: incidence, management, and outcome analysis in a tertiary care center over one decade. *European Radiology*.

[6] Hossain A., Reis E. D., Dave S. P., Kerstein M. D., Hollier L. H. (2001). Visceral artery aneurysms: experience in a tertiary-care center. *The American Surgeon*.

[7] Panayiotopoulos Y. P., Assadourian R., Taylor P. R. (1996). Aneurysms of the visceral and renal arteries. *Annals of the Royal College of Surgeons of England*.

[8] Bedford P. D., Lodge B. (1960). Aneurysm of the splenic artery. *Gut*.

[9] Arabia R., Pellicanò S., Siciliani R., Dattola O. L., Giusti S., Terra L. (1999). Splenic artery aneurysm and portal hypertension. Report of a case. *Minerva Medica*.

[10] Pasha S. F., Gloviczki P., Stanson A. W., Kamath P. S. (2007). Splanchnic artery aneurysms. *Mayo Clinic Proceedings*.

[11] Yadav R., Tiwari M. K., Mathur R. M., Verma A. K. (2009). Unusually giant splenic artery and vein aneurysm with arteriovenous fistula with hypersplenism in a nulliparous woman. *Interactive Cardiovascular and Thoracic Surgery*.

[12] Karsidag T., Soybir G., Tuzun S., Makine C. (2009). Splenic artery aneurysm rupture. *Chirurgia*.

[13] Karaman K., Onat L., Şirvancı M., Olga R. (2005). Endovascular stent graft treatment in a patient with splenic artery aneurysm. *Diagnostic and Interventional Radiology*.

[14] Sadat U., Dar O., Walsh S., Varty K. (2008). Splenic artery aneurysms in pregnancy—a systematic review. *International Journal of Surgery*.

[15] Chadha M., Ahuja C. (2009). Visceral artery aneurysms: diagnosis and percutaneous management. *Seminars in Interventional Radiology*.

[16] Miao Y.-D., Ye B. (2013). Intragastric rupture of splenic artery aneurysms: three case reports and literature review. *Pakistan Journal of Medical Sciences*.

[17] Garbagna G., Cornalba G., Rota L. (1980). Splenic artery aneurysms in patients with portal hypertension. *Radiologia Medica*.

[18] Holdsworth R. J., Gunn A. (1992). Ruptured splenic artery aneurysm in pregnancy. A review. *British Journal of Obstetrics and Gynaecology*.

[19] Bishop N. L. (1984). Splenic artery aneurysm rupture into the colon diagnosed by angiography. *British Journal of Radiology*.

[20] Tessier D. J., Stone W. M., Fowl R. J. (2003). Clinical features and management of splenic artery pseudoaneurysm: case series and cumulative review of literature. *Journal of Vascular Surgery*.

[21] Mattar S. G., Lumsden A. B. (1995). The management of splenic artery aneurysms: experience with 23 cases. *The American Journal of Surgery*.

[22] Stanley J. C., Thompson N. W., Fry W. J. (1970). Splanchnic artery aneurysms. *Archives of Surgery*.

[23] Sam C. E., Rabl M., Joura E. A. (2000). Aneurysm of the splenic artery: rupture in pregnancy. *Wiener Klinische Wochenschrift*.

[24] de Csepel J., Quinn T., Gagner M. (2001). Laparoscopic exclusion of a splenic artery aneurysm using a lateral approach permits preservation of the spleen. *Surgical Laparoscopy, Endoscopy and Percutaneous Techniques*.

[25] Selo-Ojeme D. O., Welch C. C. (2003). Review: spontaneous rupture of splenic artery aneurysm in pregnancy. *European Journal of Obstetrics & Gynecology and Reproductive Biology*.

[26] de Vries J. E., Schattenkerk M. E., Malt R. A. (1982). Complications of splenic artery aneurysm other than intraperitoneal rupture. *Surgery*.

[27] Puttini M., Aseni P., Brambilla G., Belli L. (1982). Splenic artery aneurysms in portal hypertension. *Journal of Cardiovascular Surgery*.

[28] Siablis D., Papathanassiou Z. G., Karnabatidis D., Christeas N., Katsanos K., Vagianos C. (2006). Splenic arteriovenous fistula and sudden onset of portal hypertension as complications of a ruptured splenic artery aneurysm: successful treatment with transcatheter arterial embolization. A case study and review of the literature. *World Journal of Gastroenterology*.

[29] O'Grady J. P., Day E. J., Toole A. L., Paust J. C. (1977). Splenic artery aneurysm rupture in pregnancy: a review and case report. *Obstetrics & Gynecology*.

[30] Trastek V. F., Pairolero P. C., Joyce J. W., Hollier L. H., Bernatz P. E. (1982). Splenic artery aneurysms. *Surgery*.

[31] Brockman R. S. L. (1930). Aneurysm of the splenic artery. *British Journal of Surgery*.

[32] Sendra F., Safran D. B., McGee G. (1995). A rare complication of splenic artery aneurysm: mesenteric steal syndrome. *Archives of Surgery*.

[33] Vlychou M., Kokkinis C., Stathopoulou S. (2008). Imaging investigation of a giant splenic artery aneurysm. *Angiology*.

[34] Flati G., Andrén-Sandberg Å., La Pinta M., Porowska B., Carboni M. (2003). Potentially fatal bleeding in acute pancreatitis: pathophysiology, prevention, and treatment. *Pancreas*.

[35] Bhasin D. K., Rana S. S., Sharma V. (2013). Non-surgical management of pancreatic pseudocysts associated with arterial pseudoaneurysm. *Pancreatology*.

[36] Ek E. T., Moulton C.-A., Mackay S. (2003). Catastrophic rectal bleeding from a ruptured splenic artery aneurysm. *ANZ Journal of Surgery*.

[37] Tirpude B., Bhanarkar H., Dakhore S., Surgule D. (2013). Giant splenic artery pseudo aneurysm masquerading as bleeding per rectum—a rare case. *Journal of Evolution of Medical and Dental Sciences*.

[38] Rao S., Sivina M., Willis I., Sher T., Habibnejad S. (2007). Massive lower gastrointestinal tract bleeding due to splenic artery aneurysm: a case report. *Annals of Vascular Surgery*.

[39] Taslakian B., Khalife M., Faraj W., Mukherji D., Haydar A. (2012). Pancreatitis-associated pseudoaneurysm of the splenic artery presenting as lower gastrointestinal bleeding: treatment with transcatheter embolisation. *BMJ Case Reports*.

[40] Kukliński A., Batycki K., Matuszewski W., Ostrach A., Kupis Z., Łęgowik T. (2014). Embolization of a large, symptomatic splenic artery pseudoaneurysm. *Polish Journal of Radiology*.

[41] Bretagne J. F., Heresbach D., Le Jean-Colin I. (1987). Splenic pseudoaneurysm rupture into the colon: colonoscopy before and after successful arterial embolization. *Surgical Endoscopy*.

[42] Reidy J. F., Rowe P. H., Ellis F. G. (1990). Technical report: splenic artery aneurysm embolisation—the preferred technique to surgery. *Clinical Radiology*.

[43] Mandel S. R., Jaques P. F., Sanofsky S., Mauro M. A. (1987). Nonoperative management of peripancreatic arterial aneurysms. A 10-year experience. *Annals of Surgery*.

[44] Lakin R. O., Bena J. F., Sarac T. P. (2011). The contemporary management of splenic artery aneurysms. *Journal of Vascular Surgery*.

[45] Woods M. S., Traverso L. W., Kozarek R. A., Brandabur J., Hauptmann E. (1995). Successful treatment of bleeding pseudoaneurysms of chronic pancreatitis. *Pancreas*.

[46] El Hamel A., Parc R., Adda G., Bouteloup P. Y., Huguet C., Malafosse M. (1991). Bleeding pseudocysts and pseudoaneurysms in chronic pancreatitis. *British Journal of Surgery*.

[47] Berceli S. A. (2005). Hepatic and splenic artery aneurysms. *Seminars in Vascular Surgery*.

[48] Vujic I., Andersen B. L., Stanley J. H., Gobien R. P. (1984). Pancreatic and peripancreatic vessels: embolization for control of bleeding in pancreatitis. *Radiology*.

[49] Pietrabissa A., Ferrari M., Berchiolli R. (2009). Laparoscopic treatment of splenic artery aneurysms. *Journal of Vascular Surgery*.

[50] Arca M. J., Gagner M., Heniford B. T., Sullivan T., Beven E. G. (1999). Splenic artery aneurysms: methods of laparoscopic repair. *Journal of Vascular Surgery*.

[51] Orsi M., Venturini M., Morelli F. Single-center experience in endovascular treatment of visceral artery aneurysms and pseudoaneurysms with Viabahn covered stent: technical aspects, success rate, complications and MDCT follow-up.

[52] Patel A., Weintraub J. L., Nowakowski F. S. (2012). Single-center experience with elective transcatheter coil embolization of splenic artery aneurysms: technique and midterm follow-up. *Journal of Vascular and Interventional Radiology*.

[53] Saltzberg S. S., Maldonado T. S., Lamparello P. J. (2005). Is endovascular therapy the preferred treatment for all visceral artery aneurysms?. *Annals of Vascular Surgery*.

[54] Carr S. C., Pearce W. H., Vogelzang R. L., McCarthy W. J., Nemcek A. A., Yao J. S. T. (1996). Current management of visceral artery aneurysms. *Surgery*.

